# Copolymerization of Ethylene and Vinyl Fluoride by Self-Assembled Multinuclear Palladium Catalysts

**DOI:** 10.3390/polym12071609

**Published:** 2020-07-19

**Authors:** Qian Liu, Richard F. Jordan

**Affiliations:** Department of Chemistry, The University of Chicago, 5735 South Ellis Avenue, Chicago, IL 60637, USA; lqdenom1216@gmail.com

**Keywords:** multinuclear catalyst, vinyl fluoride, copolymerization, fluorinated polyethylene

## Abstract

The self-assembled multinuclear Pd^II^ complexes {(Li-OPO^OMe2^)PdMe(4-5-nonyl-pyridine)}_4_Li_2_Cl_2_ (**C**, Li-OPO^OMe2^ = PPh(2-SO_3_Li-4,5-(OMe)_2_-Ph)(2-SO_3_^−^-4,5-(OMe)_2_-Me-Ph)), {(Zn-OP-P-SO)PdMe(L)}_4_ (**D**, L = pyridine or 4-*^t^*Bu-pyridine, [OP-P-SO]^3−^ = P(4-^t^Bu-Ph)(2-PO_3_^2−^-5-Me-Ph)(2-SO_3_^−^-5-Me-Ph)), and {(Zn-OP-P-SO)PdMe(pyridine)}_3_ (**E**) copolymerize ethylene and vinyl fluoride (VF) to linear copolymers. VF is incorporated at levels of 0.1–2.5 mol% primarily as in-chain -CH_2_CHFCH_2_- units. The molecular weight distributions of the copolymers produced by **D** and **E** are generally narrower than for catalyst **C**, which suggests that the Zn-phosphonate cores of **D** and **E** are more stable than the Li-sulfonate-chloride core of **C** under copolymerization conditions. The ethylene/VF copolymerization activities of **C**–**E** are over 100 times lower and the copolymer molecular weights (MWs) are reduced compared to the results for ethylene homopolymerization by these catalysts.

## 1. Introduction

**Figure 1:**
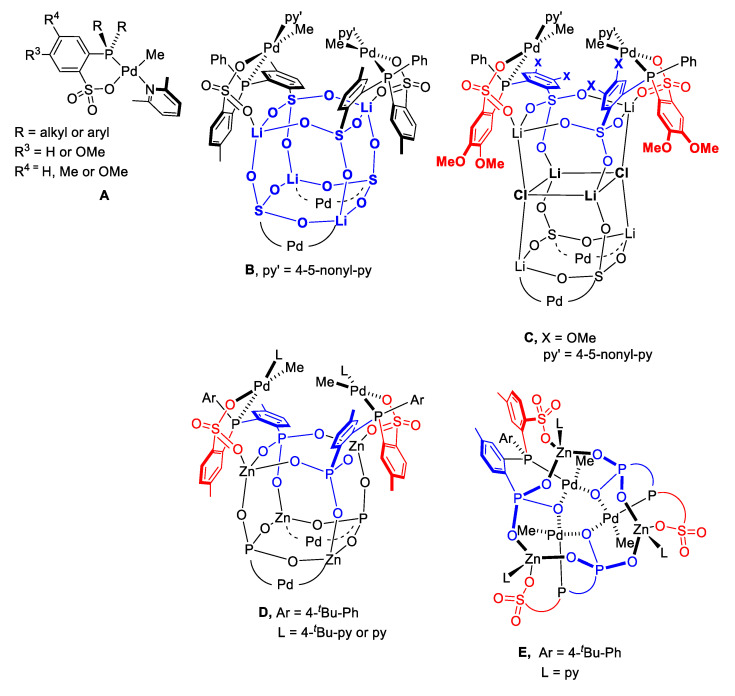
(PO)PdR Complexes. Py’ = 4-(5-nonyl)pyridine. The lower (Li-OPO)PdMe(py’) or (OP-P-SO)PdMeL units in the schematic structure of **B**, **C**, and **D** are denoted by “Pd”.

The coordination–insertion copolymerization of ethylene with polar vinyl monomers by Pd^II^ catalysts has been extensively studied [1,2,3,4,5,6,7,8]. Vinyl halides are particularly challenging polar comonomers due to (i) their poor competition with ethylene for binding to Pd^II^ catalysts [9,10], (ii) the formation of inactive L_n_Pd-X complexes by β-X elimination of L_n_PdCH_2_CXR species (generated by 1,2 CH_2_=CHX insertion or 2,1- CH_2_=CHX insertion followed by chain walking) [11,12,13,14,15,16,17,18,19,20], and (iii) the low insertion reactivity of L_n_PdCHXCH_2_R species formed by 2,1 CH_2_=CHX insertion [16,21]. We previously reported that (PO)PdMe(L) catalysts (**A**, Figure 1) that contain phosphine-arenesulfonate ligands (PO^−^) copolymerize ethylene and vinyl fluoride (VF) to linear copolymers with up to 0.55 mol% VF incorporation [22,23,24,25]. The catalyst activities are significantly reduced and the polymer molecular weights (MWs) are also reduced in ethylene/VF copolymerization compared to ethylene homopolymerization under the same conditions. For example, {P(2-Et-Ph)_2_(2-SO_3_-Ph)}PdMe(py) exhibits an activity of 296 kg∙mol^−1^∙h^−1^ in ethylene homopolymerization and produces polyethylene (PE) with *M_n_* = 16,560 Da (300 psi ethylene, toluene, 80 °C), while in ethylene/VF copolymerization the activity and polymer *M_n_* are reduced to 5 kg∙mol^−1^∙h^−1^ and 6800 Da, respectively (300 psi total pressure, VF/ethylene = 4/1, toluene, 80 °C) [22]. The ethylene/VF copolymers produced by (PO)PdMe(L) catalysts contain internal -CH_2_CHFCH_2_- units formed by 1,2 and/or 2,1 VF insertion into growing (PO)PdR species. The copolymers also contain -CH_2_CHFCH_3_, -CH_2_CHF_2_, and -CH_2_CH_2_F chain ends. The -CH_2_CHFCH_3_ units are formed by 2,1 VF insertion into (PO)PdH species followed by chain growth. It was proposed that the -CH_2_CHF_2_ and -CH_2_CH_2_F groups are generated by VF or ethylene insertion of (PO)PdF species (formed by β-F elimination of (PO)Pd(CH_2_CHFR) species), followed by chain growth. Strong support for this proposal was provided by the demonstration that (PO^BpOMe^)PdF(2,6-lutidine) ([PO^Bp,OMe^]^−^ = [P(2′,6′-(OMe)_2_-2-biphenyl)(2-OMe-Ph)(2-SO_3_-5-Me-Ph)]^-^) reacts with VF to yield the 1,2-insertion product (PO^Bp,OMe^)Pd(CH_2_CHF_2_)(2,6-lutidine) and with ethylene to yield PE with -CH_2_CH_2_F end groups [23]. It was also demonstrated that (PO^Bp,OMe^)Pd(CH_2_CHF_2_)(2,6-lutidine) reacts with ethylene to produce PE with -CH_2_CHF_2_ chain ends. The ability of (PO)PdF species to undergo olefin insertions is key to the ability of (PO)PdRL species to catalyze ethylene/VF copolymerization. The copolymerization of ethylene with other CH_2_=CHX monomers by (PO)PdRL catalysts has been extensively studied [26,27,28,29,30,31,32,33,34].

The related (Li-OPO)PdMe(py’) complex (py’ = 4-5-nonyl-pyridine) based on the phosphine-*bis*(arensulfonate) ligand PPh(2-SO_3_Li-5-Me-Ph)(2-SO_3_^−^-5-Me-Ph) (Li[OPO]^−^) self-assembles into the tetrameric {(Li-OPO)PdMe(py’)}_4_ species **B**, which is held together by a Li_4_S_4_O_12_ double 4-ring (D4R) cage (Figure 1) [35,36,37]. “Pd_4_ cage” catalyst **B** produces linear PE with a high MW and linear ethylene/VF copolymers that contain up to 3.6 mol% VF (Table 1, entries 8,9). The ethylene/VF copolymers produced by **B** contain internal -CH_2_CHFCH_2_- units as well as -CH_2_CHFCH_3_ and -CH=CHF end groups, but no NMR-detectable -CH_2_CF_2_H or -CH_2_CH_2_F chain ends. The -CH=CHF chain ends are most likely formed by 2,1 VF insertion into growing (Li-OPO)Pd-R species, followed by β-H elimination. However, **B** undergoes partial disassembly to the monomeric (Li-OPO)PdMe(py’) “Pd_1_” species under polymerization conditions, which strongly influences the MWs and molecular weight distributions (MWDs) of the polyethylene and ethylene/VF copolymers it produces. For example, **B** produces a high-MW ethylene/VF copolymer with a broad bimodal MWD (*M_w_* = 494 kDa, PDI = 310; Table 1, entry 9) in hexanes suspension, due to competing copolymerization by intact **B** (which generates the high-MW fraction) and monomeric (Li-OPO)PdR species (which generates the low-MW fraction). In contrast, **B** produces a low-MW copolymer with a narrow MWD (*M_w_* = 4200, PDI = 2.4; entry 8) in toluene solution, indicative of nearly complete dissociation to monomeric species.

**Figure 2:**
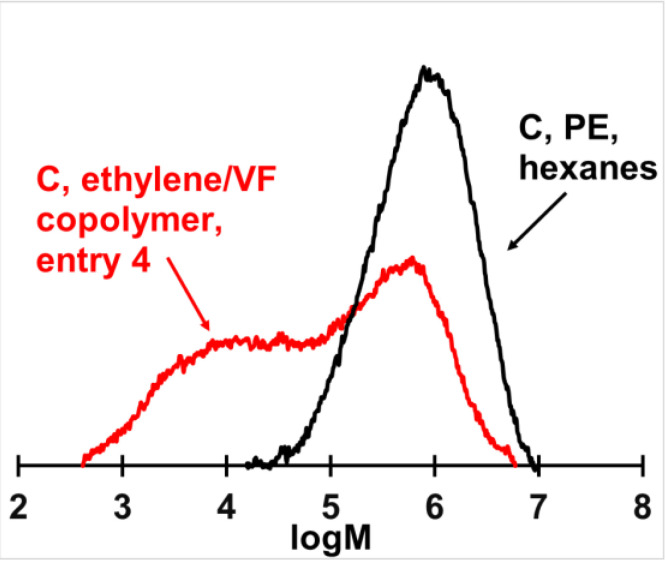
Molecular weight distributions of polyethylene (PE) [39] and ethylene/VF copolymer (Table 1, entry 4) generated by **C** determined by high temperature GPC. Polymerization conditions: hexanes solvent, 80 °C.

We recently reported several new multinuclear Pd “cage” catalysts (**C**‒**E**) [39,40]. The sterically-expanded phosphine-bis(arensulfonate) ligand PPh(2-SO_3_Li-4,5-(OMe)_2_-Ph)(2-SO_3_^−^-4,5-(OMe)_2_-Me-Ph) ([Li-OPO^OMe2^]^−^) directs the self-assembly of the tetranuclear complex (Li-OPO^OMe2^)PdMe(py’)}_4_Li_2_Cl_2_ (**C**, Figure 1), in which four (Li-OPO^OMe2^)PdMe(py’) units are arranged around the periphery of a Li_4_S_4_O_12_•Li_2_Cl_2_ cage [39]. Compound **C** is much less susceptible to cage dissociation than **B**. For example, **C** undergoes only 6.5% dissociation to monomeric species in CDCl_2_CDCl_2_ solution at 80 °C ([Pd_4_]_initial_ = 4.7 mM), whereas **B** undergoes 38% dissociation under these conditions. In toluene, **C** is only partially dissociated into monomeric species and therefore produces PE with broad MWD as expected for a multi-site catalyst. In contrast, in hexanes suspension at 80 °C, **C** is resistant to disassembly and exhibits nearly ideal single-site behavior in ethylene homopolymerization and produces high-MW PE (*M_w_* = 1473 kDa, PDI = 2.3, Figure 2). The solvent effects on the MWDs of the PEs produced by **B** and **C** may appear to be opposite but simply reflect the relative stabilities of **B** and **C** toward disassembly to monomeric species.

The phosphine-arenephosphonate-arenesulfonate ligand P(4-^t^Bu-Ph)(2-PO_3_^2−^-5-Me-Ph)(2-SO_3_^−^-5-Me-Ph) ([OP-P-SO]^3−^) directs the self-assembly of the tetrameric complex {(Zn-OP-P-SO)PdMe(L)}_4_ (**D**, Figure 1, L = py or 4-*^t^*Bu-py), which is a direct structural analogue of **B [40]**. The Zn-phosphonate interactions in the central D4R Zn_4_P_4_O_12_ cage of **D** are stronger than the Li-sulfonate and Li-chloride interactions in the cores of **B** and **C** and, as a result, **D** is more thermally stable, exhibiting no NMR-detectable dissociation to monomeric species at 80 °C in CDCl_2_CDCl_2_ solution. However, **D** (L = 4-*^t^*Bu-py) is isolated as a mixture of the major diastereomer shown in Figure 1 and ca. 20% of minor diastereomers that differ in the relative stereochemical configurations at the phosphorus centers. In the presence of 1 equiv B(C_6_F_5_)_3_ per 4-*^t^*Bu-py, **D** produces PE with a high MW but a broad MWD (*M_w_* = 436 kDa, PDI = 15; toluene/chlorobenzene solvent, 80 °C, 410 psi ethylene), consistent with the expected multi-site catalysis.

**D** (L = py) is converted to trimeric complex {(Zn-OP-P-SO)PdMe(py)}_3_ (**E**, Figure 1) in the presence of methanol. **E** adopts a cage structure composed of Zn_3_P_3_O_6_ and Pd_3_O_3_ rings linked through bridging (aryl)PO_3_^2−^ groups and is thermally stable at 80 °C in CDCl_2_CDCl_2_ solution. In the presence of 1 equiv of B(C_6_F_5_)_3_ per pyridine, **E** produces high-MW linear PE with a narrow MWD indicative of single-site catalysis (*M_w_* = 691 kDa, PDI = 2.7; toluene/chlorobenzene solvent, 80 °C, 410 psi ethylene).

The availability of these new, more stable multinuclear Pd assemblies provides an opportunity to probe the reactivity of Pd cage catalysts while reducing the complications from thermal cage disassembly. In this paper we report the ethylene/VF copolymerization behavior of **C**–**E**.

## 2. Results and Discussion

**Scheme 1:**
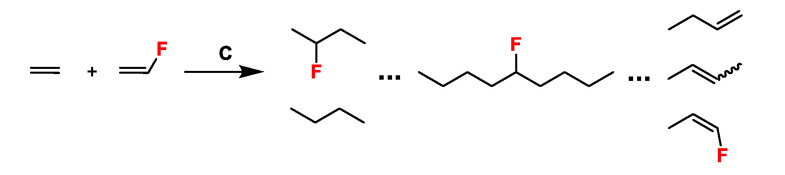
Ethylene/VF Copolymerization by Catalyst B.

**Figure 3:**
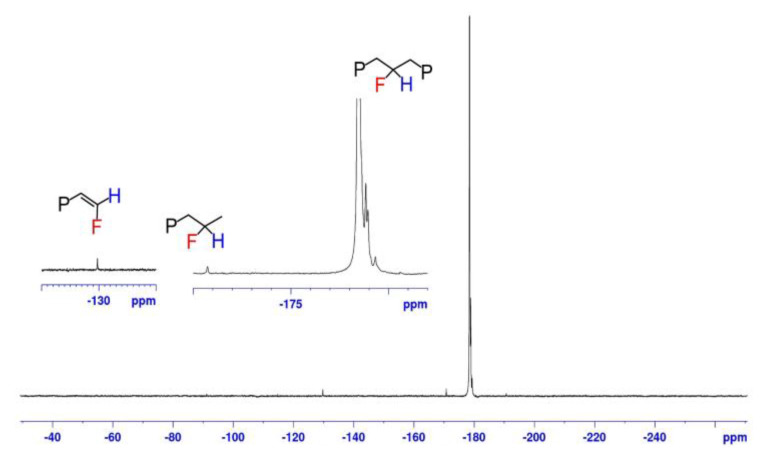
^19^F{^1^H} NMR spectrum of ethylene/VF copolymer (*o*-dichlorobenzene-*d*_4_, 120 °C) produced by **C** (Table 1, entry 4).

Ethylene/VF copolymerizations were carried out using **C** as the catalyst at 80 °C in toluene or hexanes (Scheme 1), and the results are shown in Table 1. Experimental details and copolymer characterization data are provided in the Supplementary Materials. In toluene, **C** produces low-MW copolymers with ≤0.25 mol% VF incorporation (entries 1,2). In contrast, in hexanes slurry, **C** generates a high-MW copolymer with 0.87 mol% VF incorporation at a VF/ethylene feed ratio of 0.36 (entry 3), which is increased to 2.5 mol% at a VF/ethylene feed ratio of 0.92 (entry 4). The microstructure of the copolymer produced by **C** is similar to that from **B** (Table 2, Figure 3), with major in-chain -CH_2_CHFCH_2_- units and minor VF-derived -CH_2_CFHCH_3_ and *cis*-CH=CHF end groups [41,42,43,44,45,46,47].

The ethylene/VF copolymers produced by **C** in hexanes exhibit broad MWDs (Table 1, entries 3,4; Figure 2). Given the nearly ideal single-site behavior observed in ethylene homopolymerization by **C** in hexanes, it is unlikely that the broadening of the MWD of the ethylene/VF copolymers formed in this solvent is due to thermal disassembly of the cage structure (Figure 2). One possibility is that nucleophilic Pd-F species generated by 1,2-VF insertion and *β*-F elimination react with the Li^+^ ions in the central cage, leading to the formation of new active Pd species [23,25,48,49,50,51,52]. Consistent with this proposal, -CH_2_CH_2_F and –CH_2_CHF_2_ end groups, which are formed by ethylene and VF insertion of Pd-F species in copolymerization by mononuclear catalysts **A**, are not observed in the copolymer produced by **C** (Figure 3) [23]. This process provides a potential catalyst deactivation pathway.

Ethylene/VF copolymerization by **D** (L = 4-^t^Bu-py) was investigated in a mixed toluene/chlorobenzene (49/1) solvent at a VF/ethylene feed ratio of 0.92 (Table 1, entry 5). **D** decomposes in the presence of free 4-^t^Bu-py (and other Lewis bases). Therefore, 1 equiv B(C_6_F_5_)_3_ per 4-^t^Bu-py was added to a solution of **D** in chlorobenzene prior to dilution with toluene to sequester the 4-^t^Bu-py that will be displaced by monomer, in order to minimize catalyst deactivation. Under these conditions, **D** produces a linear copolymer with 0.96 mol% VF incorporation. The MWD of the copolymer formed by **D** is unimodal but somewhat broadened (PDI = 4.1), which can be attributed to the presence of several diastereomeric forms of the catalyst. The copolymer contains in-chain -CH_2_CFHCH_2_- and a small amount of VF-derived -CH_2_CFHCH_3_ chain ends. Catalyst **E** behaves similarly to **D**, incorporating 1.1 mol% VF in the form of both -CH_2_CFHCH_2_- (major) and -CH_2_CFHCH_3_ (minor) units (Table 1, entry 7). The MWDs of the copolymers formed by **E** are narrow (PDI ≤ 2.4), indicative of nearly ideal single-site catalysis. These results suggest that the multinuclear structures of **D** and **E** are substantially retained during ethylene/VF copolymerization.

The ethylene/VF copolymerization activities of **C**–**E** are over 100 times lower than the ethylene homopolymerization activities, and the copolymer MWs are reduced compared to the results for ethylene homopolymerization, as observed previously for **A** and **B** and mononuclear (PO)PdRL catalysts.

## 3. Conclusions

“Pd cage” catalysts **C**‒**E** copolymerize ethylene with VF to linear copolymers with 0.1 to 2.5 mol% VF incorporation depending on the catalyst and reaction conditions. VF is incorporated predominantly as in-chain -CH_2_CHFCH_2_- units, with minor -CH_2_CFHCH_3_ and, for **C,** *cis*-CH=CHF end groups. **C** produces an ethylene/VF copolymer with a broad MWD in hexanes, which contrasts results for ethylene homopolymerization where nearly ideal single-site behavior is observed. These results suggest that the presence of VF promotes structural disruption of the catalyst, possibly through reaction of Pd-F species with the Li^+^ ions in the cage. The ethylene/VF copolymerization behavior of **C** is very similar to that of **B**, except the extent of VF incorporation by **C** is somewhat lower than by **B**. **D** and **E** produce ethylene/VF copolymers with narrow MWDs, suggesting that the cage assemblies remain substantially intact during copolymerization. The activities of **C**–**E** in ethylene/VF copolymerization are >100 times lower than in ethylene homopolymerization. This inhibition effect is a major roadblock to the development of more practical ethylene/VF copolymerization catalysts.

## Figures and Tables

**Table 1:** Ethylene/vinyl-fluoride copolymerization by catalysts **C**, **D,** and **E** [38].

Entry	Cat.	Solvent	*P*_C2H4_ (psi)	*P*_VF_ (psi)	Acitivity *^d^* (kg∙mol^−1^∙h^−1^)	M_w_ *^e^* (10^3^)	PDI *^e^*	VF Incorp *^f^* (mol%)	T_m_ *^g^* (°C)
1 *^a^*	**C**	toluene	220	80	1.4	20.3	8.0	0.10	128.8
2 *^a^*	**C**	toluene	130	120	0.44	1.92	1.4	0.25	ND *^h^*
3 *^a^*	**C**	hexanes	220	80	12.0	498	18.2	0.87	134.4
4 *^a^*	**C**	hexanes	130	120	3.4	419	26.2	2.5	132.8
5 *^a,b^*	**D**	toluene + PhCl	130	120	2.0	42.2	4.1	0.96	131.4
6 *^a,b^*	**E**	toluene + PhCl	220	80	4.9	50.3	2.4	0.43	134.7
7 *^a,b^*	**E**	toluene + PhCl	130	120	1.0	23.0	2.3	1.1	131.6
8 *^a,c^*	**B**	toluene	130	120	1.9	4.2	2.4	2.4	127.8
9 *^a,c^*	**B**	hexanes	130	120	1.4	494	310	3.6	127.8

*^a^* Conditions: [Pd] = 10 μmol, temperature = 80 °C, time = 2 h, 50 mL solvent. *^b^* 1 equiv B(C_6_F_5_)_3_ per L, 49/1 toluene/chlorobenzene solvent. ^*c*^ cited from reference 36. ^*d*^ Activity is reported per mol Pd. ^*e*^ Determined by Gel Permeation Chromatography (GPC) [38]. *^f^* VF incorporation in copolymer determined by ^1^H NMR. ^g^ DSC. ^h^ Not determined due to the limited quantity of copolymer.

**Table 2:** Microstructures of E/VF copolymers produced by catalysts **C**, **D,** and **E [41,42,43,44,45,46,47]**.

Catalyst	Entry in Table 1	Distribution of VF Incorporation Modes (%) *^a^*		
		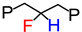	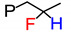	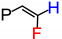
**C**	1	100	Not observed	Not observed
**C**	2	85.1	5.0	9.9
**C**	3	99.1	0.9	Not observed
**C**	4	99.2	0.4	0.4
**D**	5	>99	Trace *^b^*	Not observed
**E**	6	99.2	0.8	Not observed
**E**	7	98.8	1.2	Not observed

*^a^* Determined by ^19^F NMR spectroscopy; -CH_2_CH_2_F and -CH_2_CHF_2_ end groups were not observed. *^b^* Resonance observed but intensity too low for accurate integration.

## Supplementary Materials

The following are available online at https://www.mdpi.com/2073-4360/12/7/1609/s1.
