# Supplementary material for: Copolymerization of Ethylene and Vinyl Fluoride by Self-Assembled Multinuclear Palladium Catalysts
Source: Polymers (Basel). 2020 Jul 19;12(7):1609. doi: 10.3390/polym12071609 (PMC7407146; doi:10.3390/polym12071609)
Supplement: Supplementary file 1 [file polymers-12-01609-s001.pdf]

Supporting Information for:

Copolymerization of Ethylene and Vinyl Fluoride by Self-assembled Multinuclear Palladium Catalysts

Qian Liu and Richard F. Jordan*

Department of Chemistry, The University of Chicago, 5735 South Ellis Avenue, Chicago, Illinois 60637, United States

Contents

I. General Procedures S-2

II. Polymerization procedures and Results S-4

III. NMR Spectra of Copolymers S-12

IV. References S-19

I. General Procedures.

All experiments were performed under a nitrogen atmosphere using drybox or Schlenk techniques. Nitrogen was purified by passage through Q-5 oxygen scavenger and activated molecular sieves. *o*-Dichlorobenzene-d_4 was purchased from Cambridge Isotope Laboratories and used as received. Hexanes, toluene and chlorobenzene were purified by passage through activated alumina and BASF R3-11 oxygen scavenger. Ethylene (polymer-grade) and vinyl fluoride (VF) were purchased from Matheson. The compounds **C** – **E** were prepared by literature procedures [1-2].

NMR spectra were acquired on Bruker DRX-500 or Bruker DRX-400 spectrometers at ambient temperatures unless otherwise indicated. ^{1}H chemical shifts are reported relative to $SiMe_4$ and are internally referenced to residual ^{1}H resonances of *o*-dichlorobenzene-d_4 solvent. $^{19}F\{^{1}H\}$ spectra were referenced to external $BF_3{\bullet}Et_2O$ and ^{19}F chemical shifts are reported relative to $CFCl_3$. ^{1}H and ^{19}F NMR spectra of polymers were obtained as follows. An NMR tube containing a mixture of polymer and *o*-dichlorobenzene-d_4 (0.5 mL) was heated to 100 °C (^{19}F NMR) or 120 °C (^{1}H NMR) to afford a homogeneous solution. The tube was inserted into a pre-heated NMR probe (DRX 500 for ^{19}F; DRX 400 for ^{1}H) and NMR spectra were obtained after a 5 min temperature equilibration period.

Gel permeation chromatography (GPC) data were obtained on a Polymer Laboratories PL-GPC 220 instrument at 150 °C with 1,2,4-trichlorobenzene (stabilized with 125 ppm BHT) as the mobile phase. Three PLgel 10 μm Mixed-B LS columns were used. Molecular weights were calibrated using narrow polystyrene standards (ten-point calibration with M_n from 570 Da to 5670 kDa) and are corrected for linear polyethylene by universal calibration using the following Mark-Houwink parameters: polystyrene, $K = 1.75 \times 10^{-2}\ cm^3g^{-1}$, $\alpha = 0.67$; polyethylene, $K = 5.90 \times 10^{-2}$

$cm^3 g^{-1}$, $\alpha = 0.69$ [3]. DSC measurements were performed on a TA Instruments DSC 2920 instrument. DSC samples (10 mg) were annealed by heating to 170 °C at 20 °C/min, cooled to 40 °C at 20 °C/min, and then analyzed while being heated to 170 °C at 20 °C/min.

II. Polymerization procedures and Results

Ethylene/VF Copolymerization. Polymerization reactions were performed in a Parr 300 mL stainless steel autoclave, which was equipped with a mechanical stirrer, thermocouple and water cooling loop and controlled by a Parr 4842 controller. In the glove box, the catalyst (10 µmol) was weighed into a glass autoclave liner. Solvent (50 mL) was added. The liner was placed in the autoclave, and the autoclave was assembled and brought out of the box. The autoclave was pressurized with VF to the desired pressure and ethylene was added until the total pressure reached 250 or 300 psi, while stirring (100 rpm). The reactor was heated to the desired temperature (80 °C) and stirring was increased to 170 rpm. After 2 h, the autoclave was cooled to 25 °C and vented. Acetone (50 mL) was added to precipitate the polymer. The polymer was collected by filtration, rinsed with acetone, and dried under vacuum.

Figure S-1. GPC analysis of ethylene/VF copolymer produced by **C** in at 80 °C for 2 h (Table 1, entry 1). Note: The acquired and last calibrated dates are incorrect due to an incorrect instrument computer setting.

Polymer Laboratories
PL Caliber GPC Software

22:02 Mon Aug 30 1999

Unknown QL4487.E00
>PE

Acquired : 21:47 Mon Aug 30 1999
Operator Richard F. Jordan

Concentration :
Injection Volume :
Solvent :
Column Set :

Detector :
Temperature :
Flow Rate : 1.000
Standards :

Method 1
Comments :

Calibration Using : Narrow Standards   Curve Used : 3rd Order Polynomial
Calibration Limits : 14.78 to 24.50 Mins   Last Calibrated : Mon Aug 30 21:54:20 1999
Flow Rate Marker : found at : Not Found   in Standards at : 0.00 Mins

Broad Peak Start : 16.12 End : 24.50 Mins

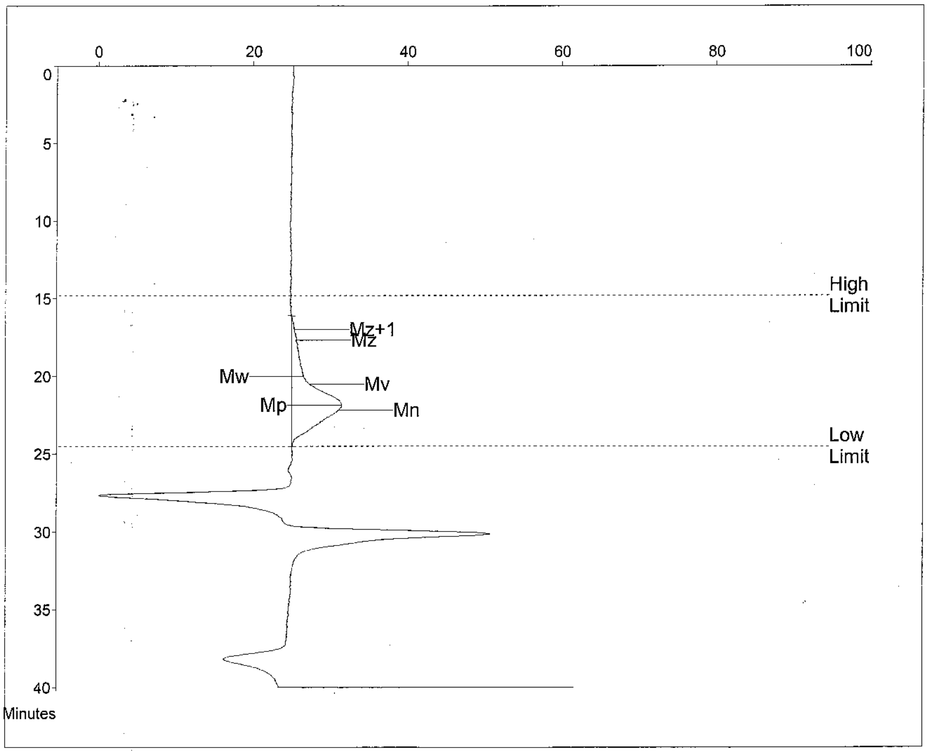

Molecular Weight Averages

Mp =	3458	Mz =	184608
Mn =	2528	Mz+1 =	350020
Mw =	20259	Mv =	12661
Polydispersity =	8.013	Peak Area =	225717

Figure S-2. GPC analysis of ethylene/VF copolymer produced by **C** in toluene at 80 ˚C for 2 h (Table 1, entry 2). Note: The acquired and last calibrated dates are incorrect due to an incorrect instrument computer setting.

Polymer Laboratories
PL Caliber GPC Software

22:02 Mon Aug 30 1999

Unknown QL4484.E00
>PE

Acquired : 21:47 Mon Aug 30 1999
Operator Richard F. Jordan

Concentration :
Injection Volume :
Solvent :
Column Set :

Detector :
Temperature :
Flow Rate : 1.000
Standards :

Method 1
Comments :

Calibration Using : Narrow Standards
Calibration Limits : 14.78 to 24.50 Mins
Flow Rate Marker : found at : Not Found

Curve Used : 3rd Order Polynomial
Last Calibrated : Mon Aug 30 21:54:20 1999
in Standards at : 0.00 Mins

Broad Peak Start : 20.98 End : 24.27 Mins

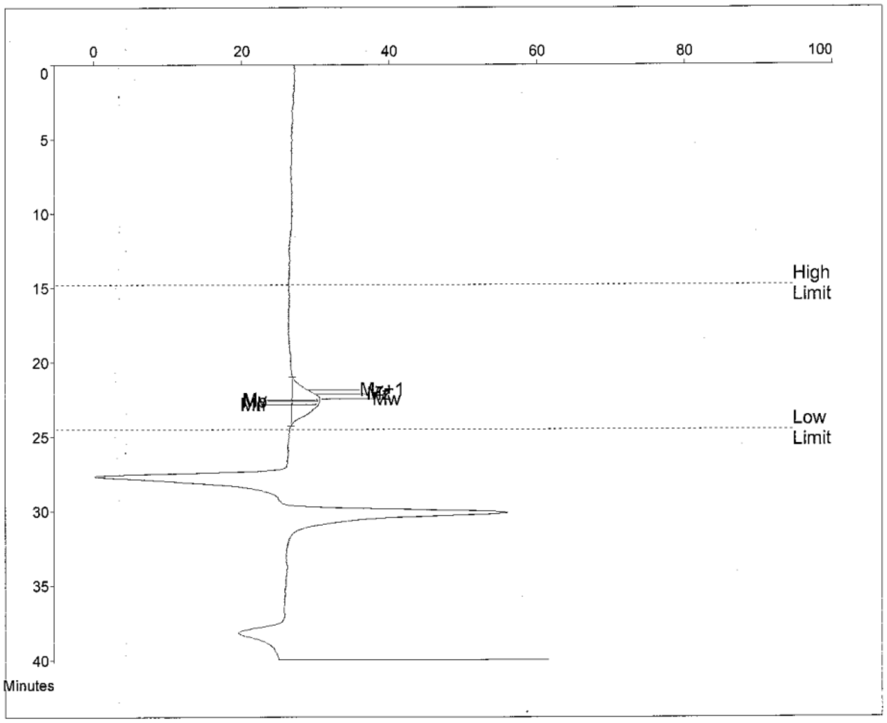

Molecular Weight Averages

Mp =	1678	Mz =	2670
Mn =	1343	Mz+1 =	3455
Mw =	1922	Mv =	1819
Polydispersity =	1.430	Peak Area =	81180

Figure S-3. GPC analysis of ethylene/VF copolymer produced by **C** in hexanes slurry at 80 ˚C for 2 h (Table 1, entry 3). Note: The acquired and last calibrated dates are incorrect due to an incorrect instrument computer setting.

Polymer Laboratories
PL Caliber GPC Software

2 day shake

21:56 Tue Aug 31 1999

Unknown QL4478.E00
>PE

Acquired : 21:50 Tue Aug 31 1999
Operator Richard F. Jordan

Concentration :
Injection Volume :
Solvent :
Column Set :

Detector :
Temperature :
Flow Rate : 1.000
Standards :

Method 1
Comments :

Calibration Using : Narrow Standards
Curve Used : 3rd Order Polynomial
Calibration Limits : 14.78 to 24.50 Mins
Last Calibrated : Tue Aug 31 21:55:04 1999
Flow Rate Marker : found at : Not Found
in Standards at : 0.00 Mins

Broad Peak Start : 14.13 End : 22.87 Mins

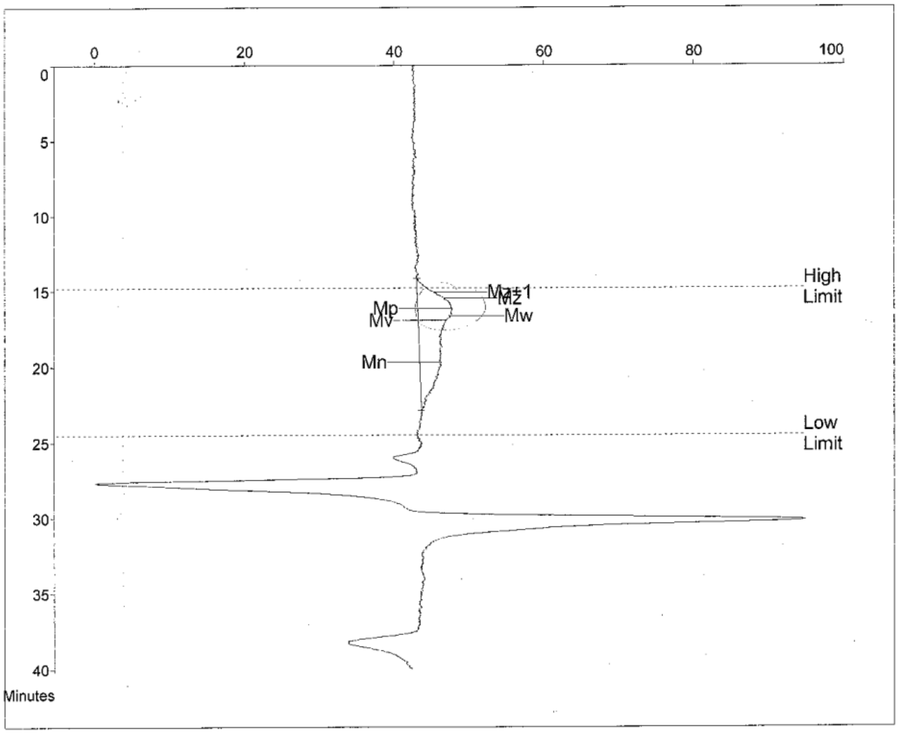

Molecular Weight Averages

Mp =	807933	Mz =	1476268
Mn =	27431	Mz+1 =	2180003
Mw =	498167	Mv =	378412
Polydispersity =	18.161	Peak Area =	261787

Figure S-4. GPC analysis of ethylene/VF copolymer produced by **C** in hexanes slurry at 80 ˚C for 2 h (Table 1, entry 4). Note: The acquired and last calibrated dates are incorrect due to an incorrect instrument computer setting.

2d shake

Polymer Laboratories
PL Caliber GPC Software

21:57 Tue Aug 31 1999

Unknown QL4483.E00
>PE

Acquired : 21:50 Tue Aug 31 1999
Operator Richard F. Jordan

Concentration :
Injection Volume :
Solvent :
Column Set :

Detector :
Temperature :
Flow Rate : 1.000
Standards :

Method 1
Comments :

Calibration Using : Narrow Standards
Calibration Limits : 14.78 to 24.50 Mins
Flow Rate Marker : found at : Not Found

Curve Used : 3rd Order Polynomial
Last Calibrated : Tue Aug 31 21:55:04 1999
in Standards at : 0.00 Mins

Broad Peak Start : 14.07 End : 23.33 Mins

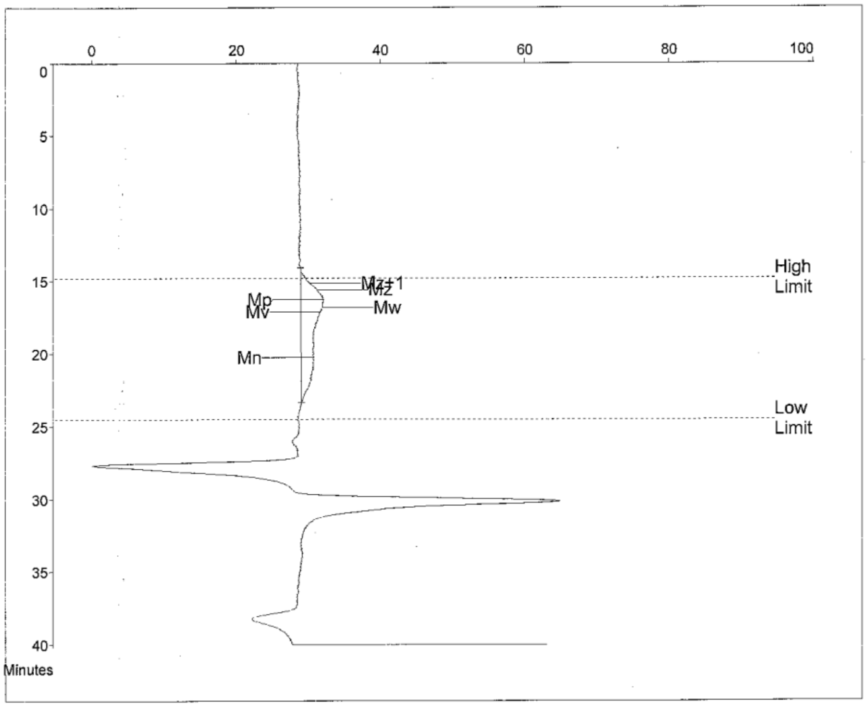

Molecular Weight Averages

Mp =	700805	Mz =	1332846
Mn =	15990	Mz+1 =	2023697
Mw =	418593	Mv =	310934
Polydispersity =	26.178	Peak Area =	186075

Figure S-5. GPC analysis of ethylene/VF copolymer produced by **D** in toluene/chlorobenzene at 80 ˚C for 2 h (Table 1, entry 5).

Polymer Laboratories
PL Caliber GPC Software

21:51 Thu Sep 01 2016

Unknown QL4501.E00
>PE

Acquired : 21:40 Thu Sep 01 2016
Operator Richard F. Jordan

Concentration :
Injection Volume :
Solvent :
Column Set :

Detector :
Temperature :
Flow Rate : 1.000
Standards :

Method 1
Comments :

Calibration Using : Narrow Standards
Calibration Limits : 14.70 to 24.40 Mins
Flow Rate Marker : found at : Not Found

Curve Used : 3rd Order Polynomial
Last Calibrated : Thu Sep 01 21:45:42 2016
in Standards at : 0.00 Mins

Broad Peak Start : 16.48 End : 23.75 Mins

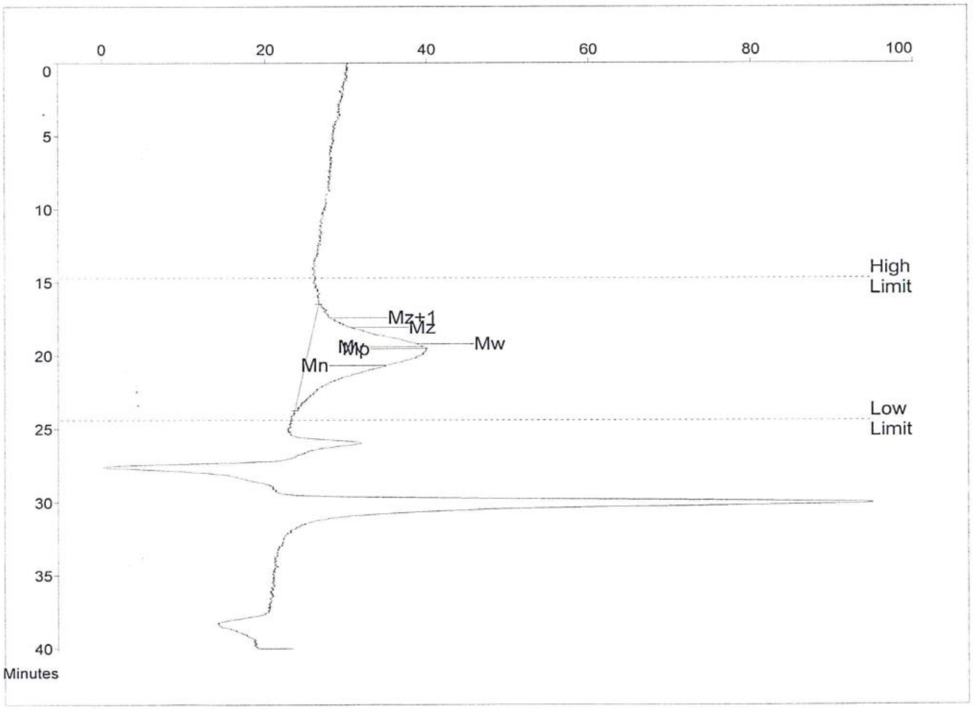

Molecular Weight Averages

Mp =	30386	Mz =	119276
Mn =	10267	Mz+1 =	225781
Mw =	42244	Mv =	35086
Polydispersity =	4.115	Peak Area =	532503

Figure S-6. GPC analysis of ethylene/VF copolymer produced by **E** in toluene/chlorobenzene at 80 ˚C for 2 h (Table 1, entry 6).

Polymer Laboratories
PL Caliber GPC Software

04:28 Fri Jul 29 2016

Unknown QL4492.E00
>Pe

Acquired : 04:20 Fri Jul 29 2016
Operator Richard F. Jordan

Concentration :
Injection Volume :
Solvent :
Column Set :

Detector :
Temperature :
Flow Rate : 1.000
Standards :

Method 1
Comments :

Calibration Using : Narrow Standards
Calibration Limits : 14.78 to 24.50 Mins
Flow Rate Marker : found at : Not Found

Curve Used : 3rd Order Polynomial
Last Calibrated : Fri Jul 29 04:25:36 2016
in Standards at : 0.00 Mins

Broad Peak Start : 16.70 End : 22.52 Mins

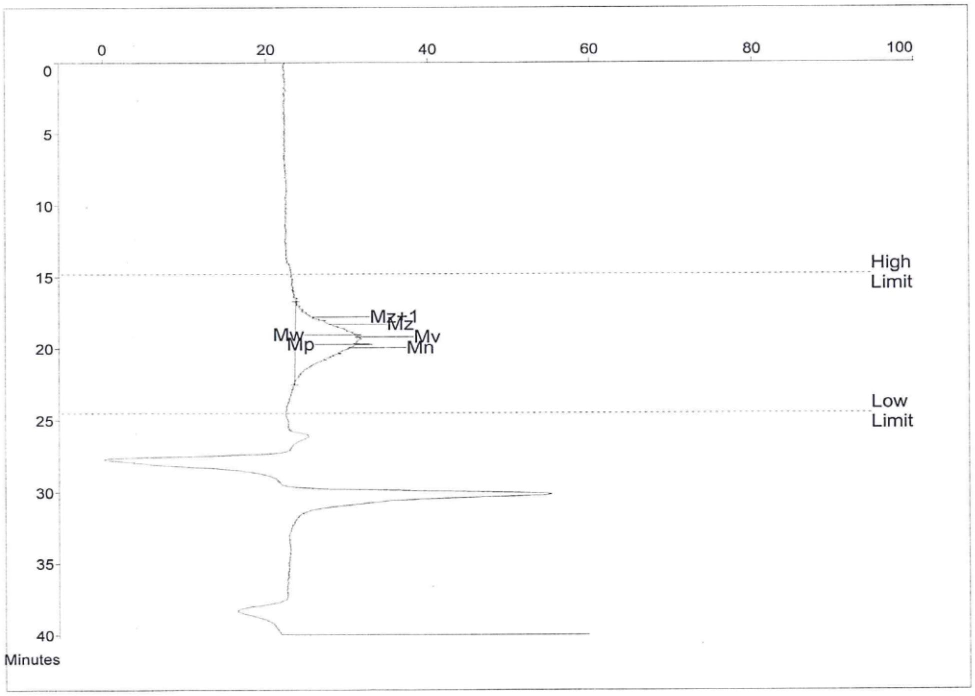

Molecular Weight Averages

Mp =	26575	Mz =	101806
Mn =	21298	Mz+1 =	164715
Mw =	50305	Mv =	44440
Polydispersity =	2.362	Peak Area =	250328

Figure S-7. GPC analysis of ethylene/VF copolymer produced by **D** in toluene/chlorobenzene at 80 ˚C for 2 h (Table 1, entry 7).

Polymer Laboratories
PL Caliber GPC Software

04:28 Fri Jul 29 2016

Unknown QL4491.E00
>PE

Acquired : 04:20 Fri Jul 29 2016
Operator Richard F. Jordan

Concentration :
Injection Volume :
Solvent :
Column Set :

Detector :
Temperature :
Flow Rate : 1.000
Standards :

Method 1
Comments :

Calibration Using : Narrow Standards
Calibration Limits : 14.78 to 24.50 Mins
Flow Rate Marker : found at : Not Found

Curve Used : 3rd Order Polynomial
Last Calibrated : Fri Jul 29 04:25:36 2016
in Standards at : 0.00 Mins

Broad Peak Start : 17.82 End : 23.17 Mins

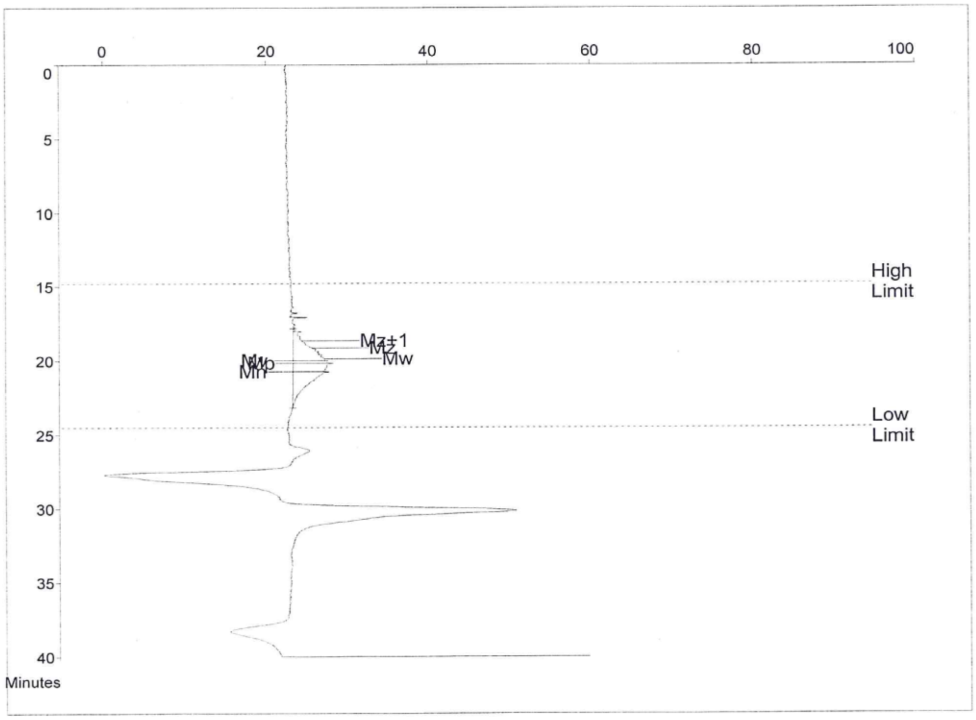

Molecular Weight Averages

Mp =	17314	Mz =	45752
Mn =	10034	Mz+1 =	71532
Mw =	22981	Mv =	20352
Polydispersity =	2.290	Peak Area =	131672

III. NMR Spectra of Copolymers

Table S-1. NMR assignments for VF units in the ethylene/VF copolymer

^{1}H NMR	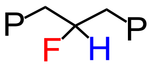	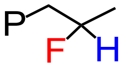	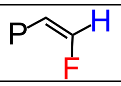
δ	4.40	4.51	6.31
J_{HF}	48.4	40.0	84.0

^{19}F{^{1}H} NMR	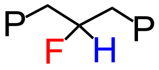	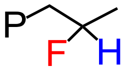	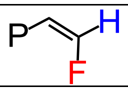
δ	-178.5	-170.7	-129.7

Figure S-8. NMR spectra of ethylene/VF copolymer produced by C (Table 1, entry 1)

(a) ^{1}H (*o*-dichlorobenzene-d_4, 120 ˚C, 400 MHz):

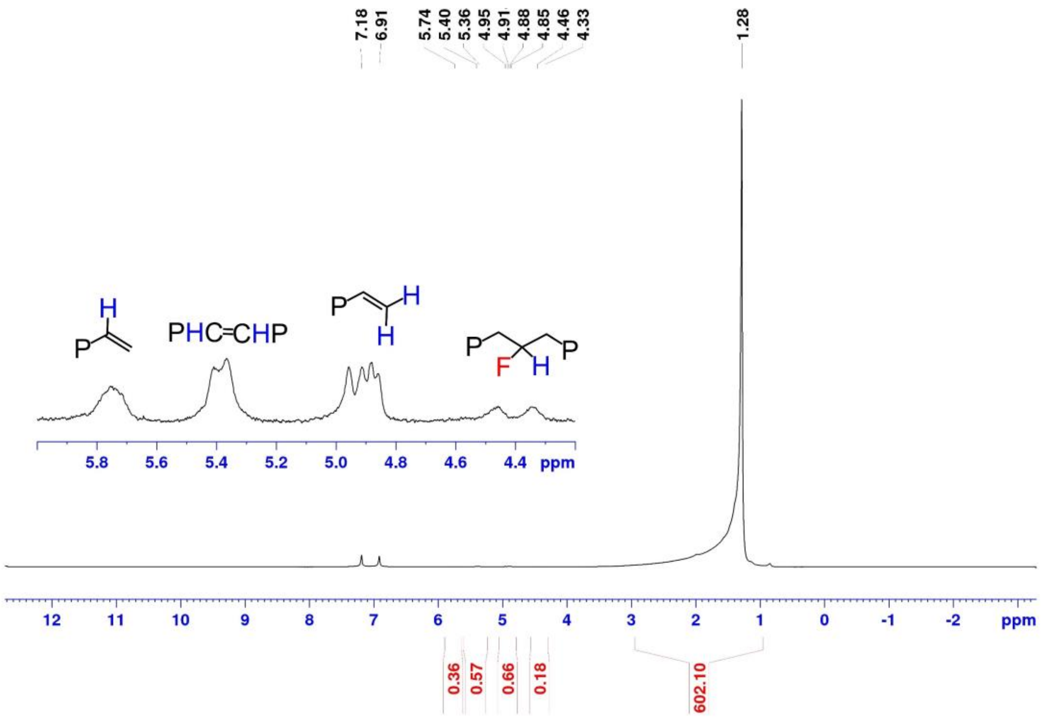

(b) $^{19}F\{^1H\}$ (*o*-dichlorobenzene-d_4, 100 ˚C, 470 MHz):

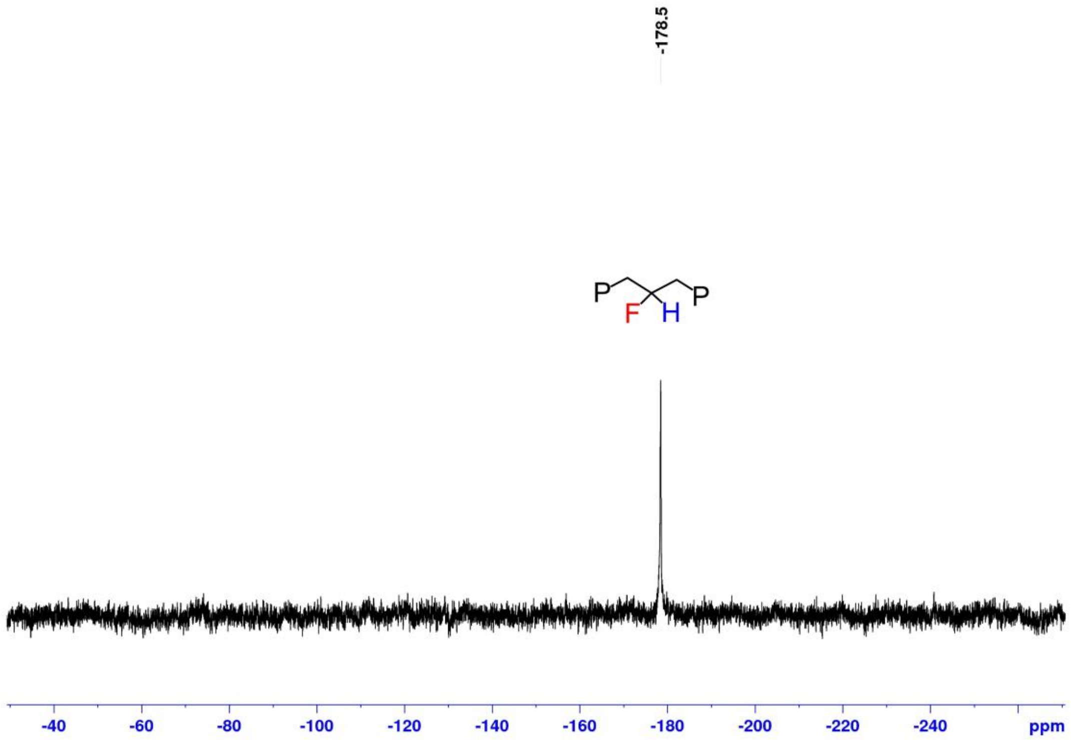

Figure S-9. NMR spectra of ethylene/VF copolymer produced by C (Table 1, entry 2)

(a) ^1H (*o*-dichlorobenzene-d_4, 120 ˚C, 400 MHz):

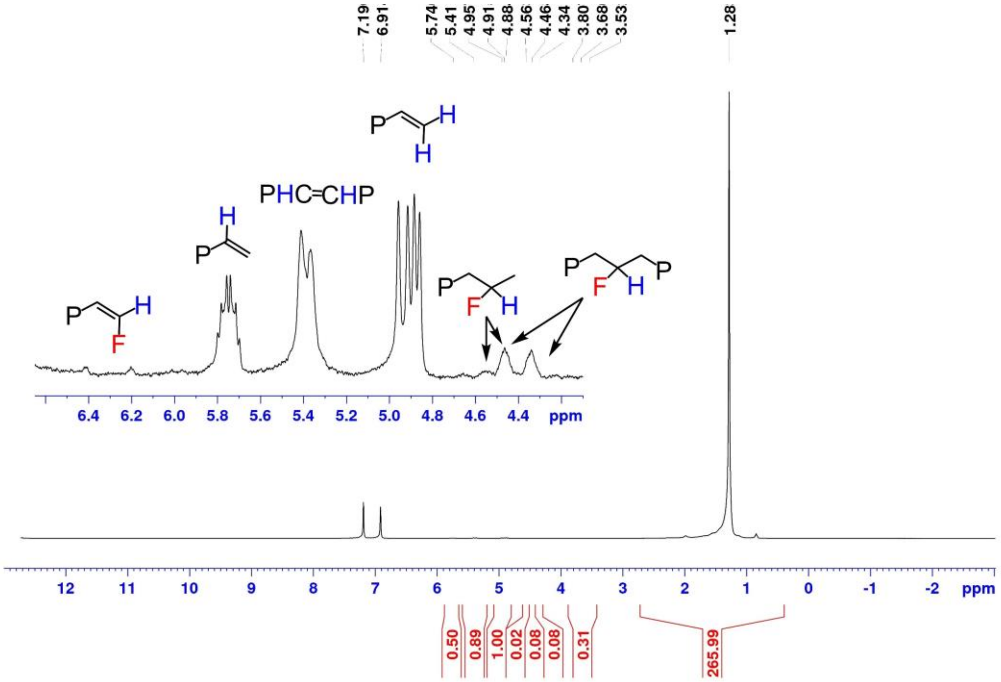

(b) $^{19}F\{^1H\}$ (*o*-dichlorobenzene-d_4, 100 ˚C, 470 MHz):

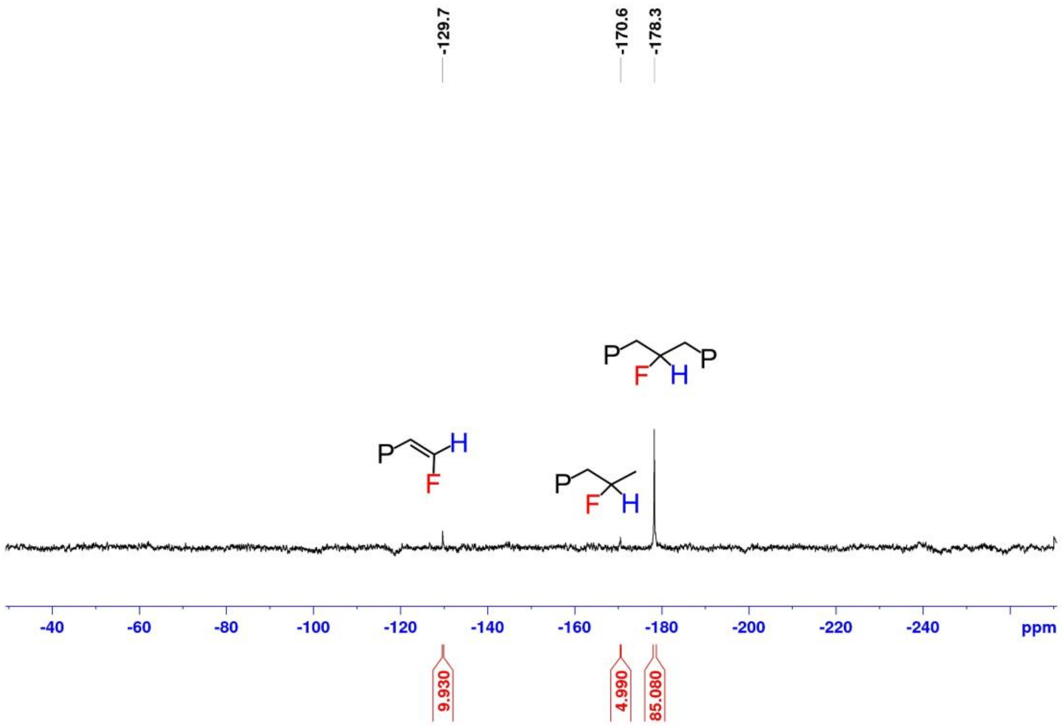

Figure S-10. NMR spectra of ethylene/VF copolymer produced by C (Table 1, entry 3)

(a) ^1H (*o*-dichlorobenzene-d_4, 120 ˚C, 400 MHz):

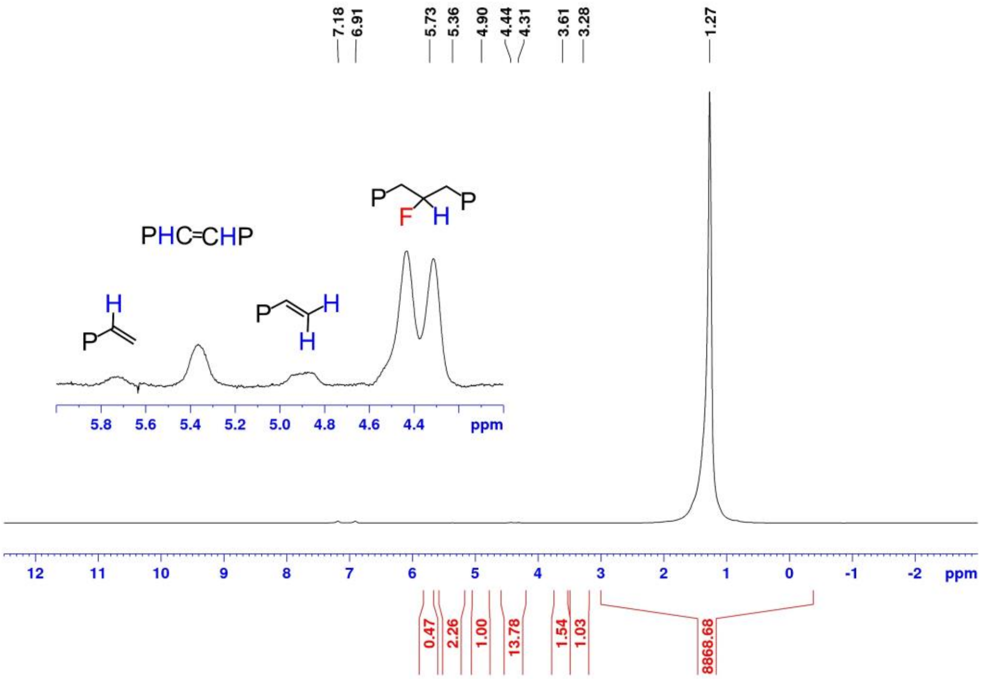

(b) $^{19}F\{^{1}H\}$ (*o*-dichlorobenzene-d_4, 100 ˚C, 470 MHz):

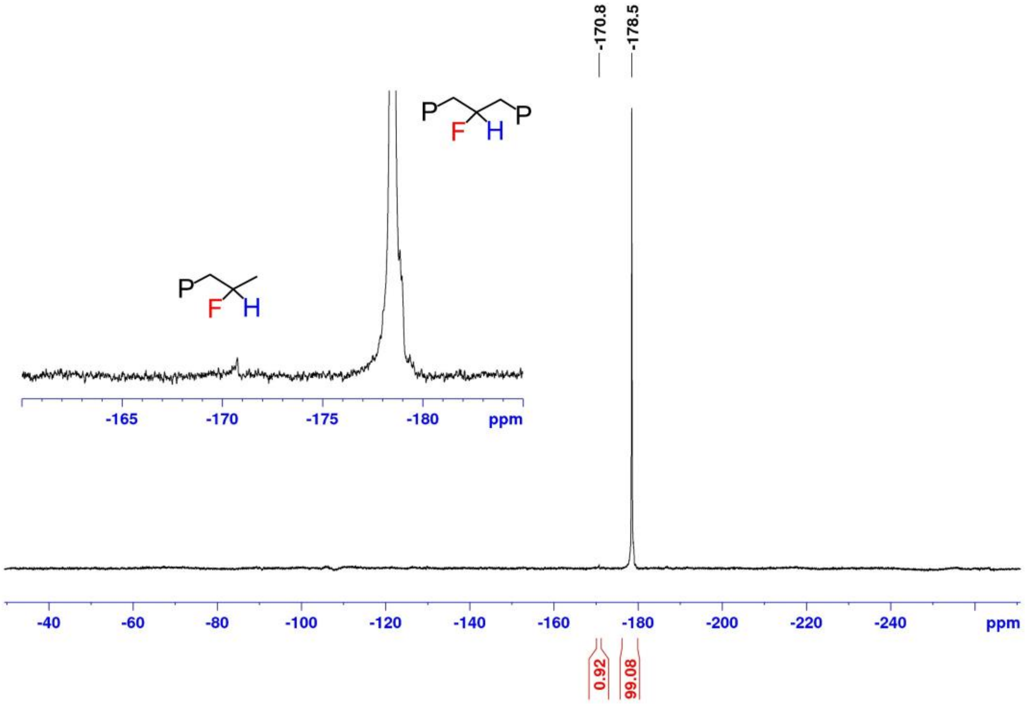

Figure S-11. NMR spectra of ethylene/VF copolymer produced by C (Table 1, entry 4)

(a) ^{1}H (*o*-dichlorobenzene-d_4, 120 ˚C, 400 MHz):

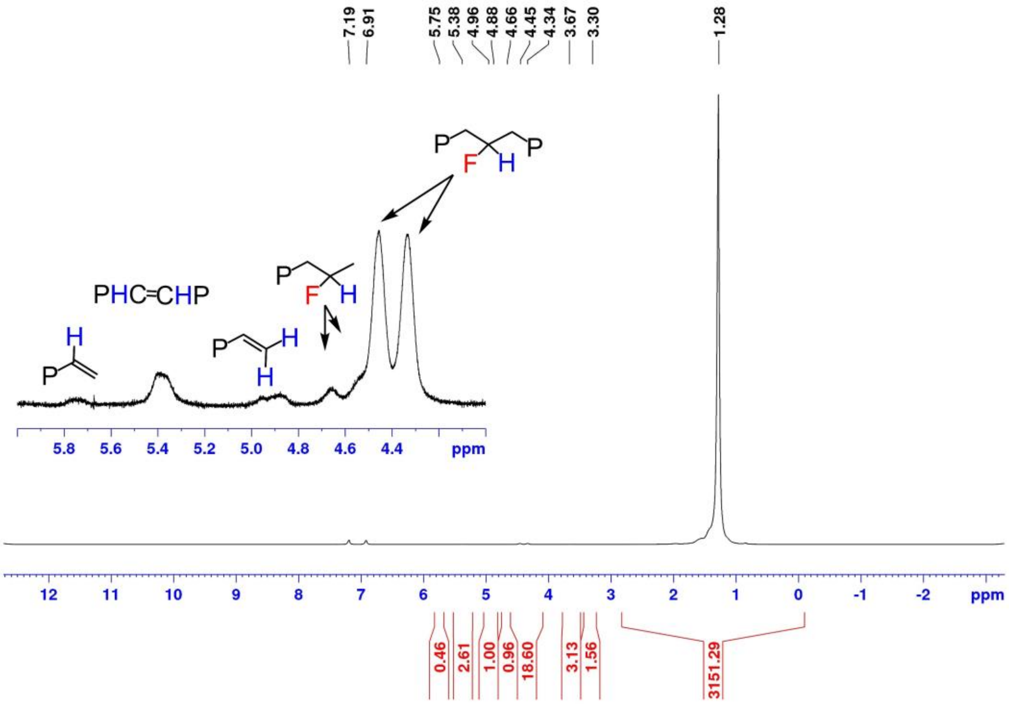

(b) $^{19}F\{^{1}H\}$ (*o*-dichlorobenzene-*d*$_4$, 100 ˚C, 470 MHz):

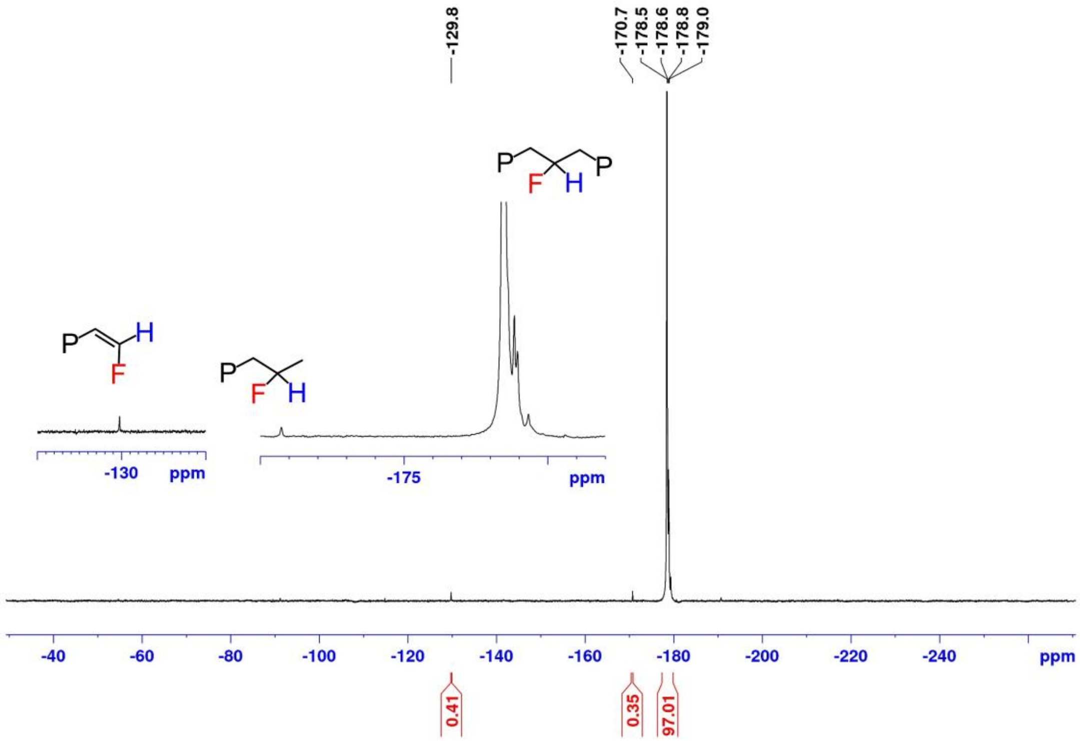

Figure S-12. NMR spectra of ethylene/VF copolymer produced by D (Table 1, entry 5)

(a) ^{1}H (*o*-dichlorobenzene-*d*$_4$, 120 ˚C, 400 MHz):

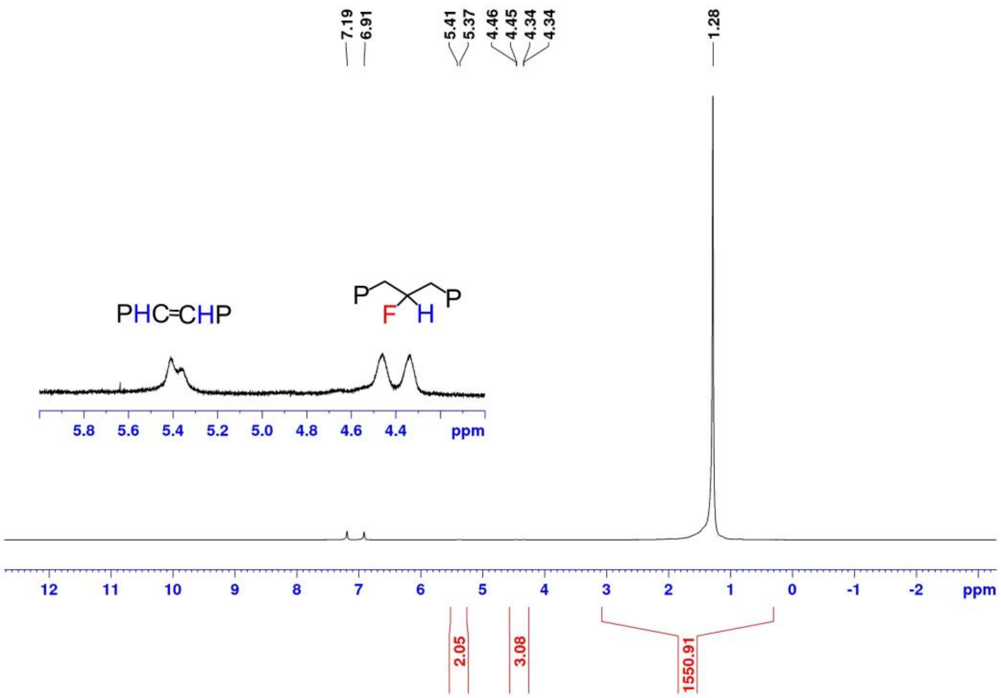

(b) $^{19}F\{^1H\}$ (*o*-dichlorobenzene-d_4, 100 ˚C, 470 MHz): (4-^{t}Bu-py)B(C_6F_5)$_3$: δ -133.9, -158.6, -161.8 [4]; -153.5: fluorosilicate species [5-7]; other peaks in the range δ -130 to -168 are ascribed to fluoride salts [8-9] and B(C_6F_5)$_3$ degradation products.

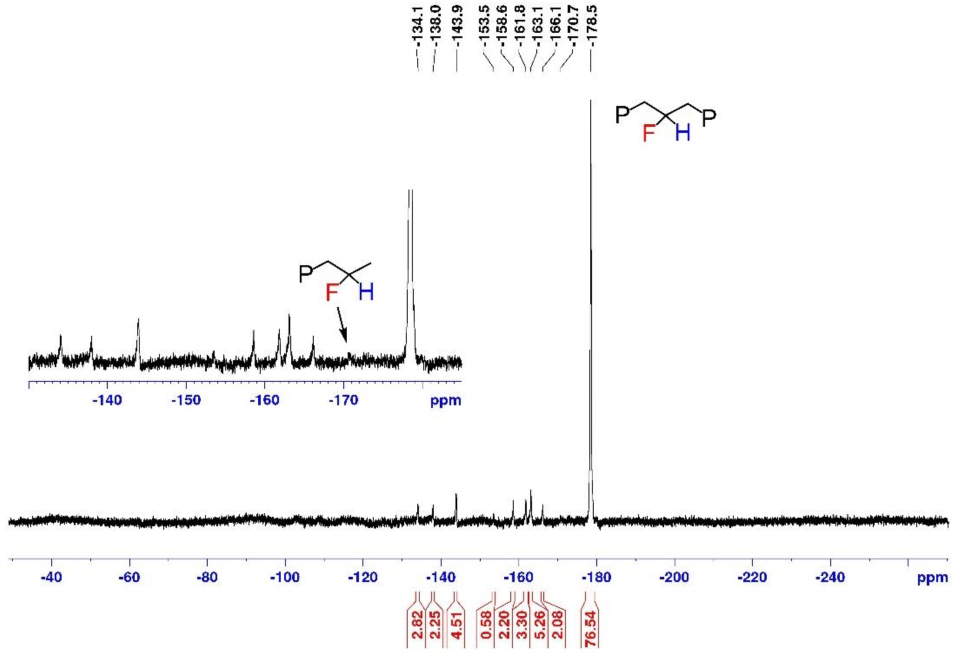

Figure S-13. NMR spectra of ethylene/VF copolymer produced by E (Table 1, entry 6)

(a) ^1H (*o*-dichlorobenzene-d_4, 120 ˚C, 400 MHz):

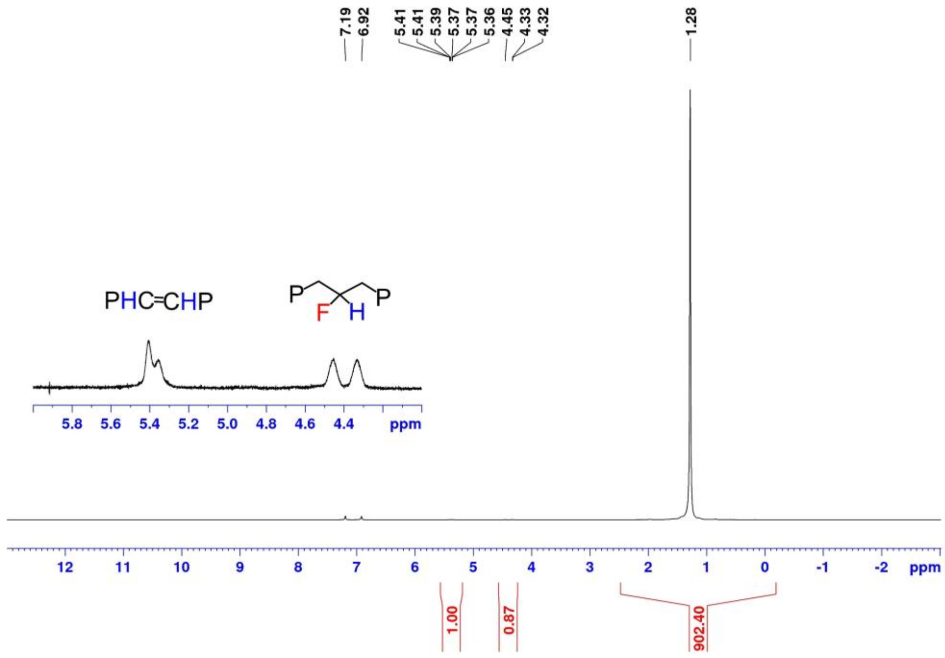

(b) $^{19}F\{^{1}H\}$ (*o*-dichlorobenzene-*d*$_4$, 100 ˚C, 470 MHz): (py)B(C_6F_5)$_3$: δ -134.2, -158.6, -163.0 [10]; -153.5: fluorosilicate species [5-7]; other peaks in the range δ -130 to -168 are ascribed to fluoride salts [8-9] and B(C_6F_5)$_3$ degradation products.

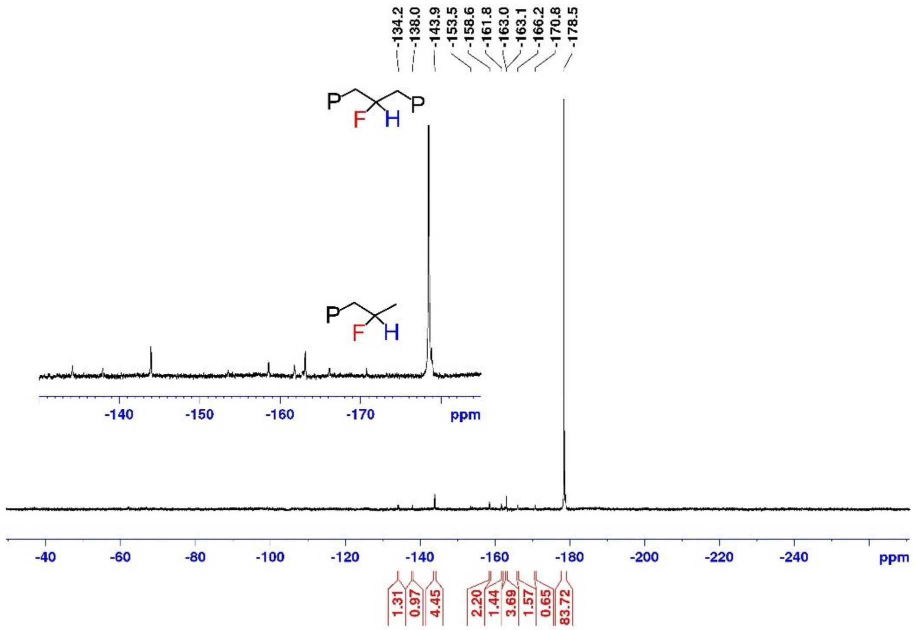

Figure S-14. NMR spectra of ethylene/VF copolymer produced by E (Table 1, entry 7)

(a) ^{1}H (*o*-dichlorobenzene-*d*$_4$, 120 ˚C, 400 MHz):

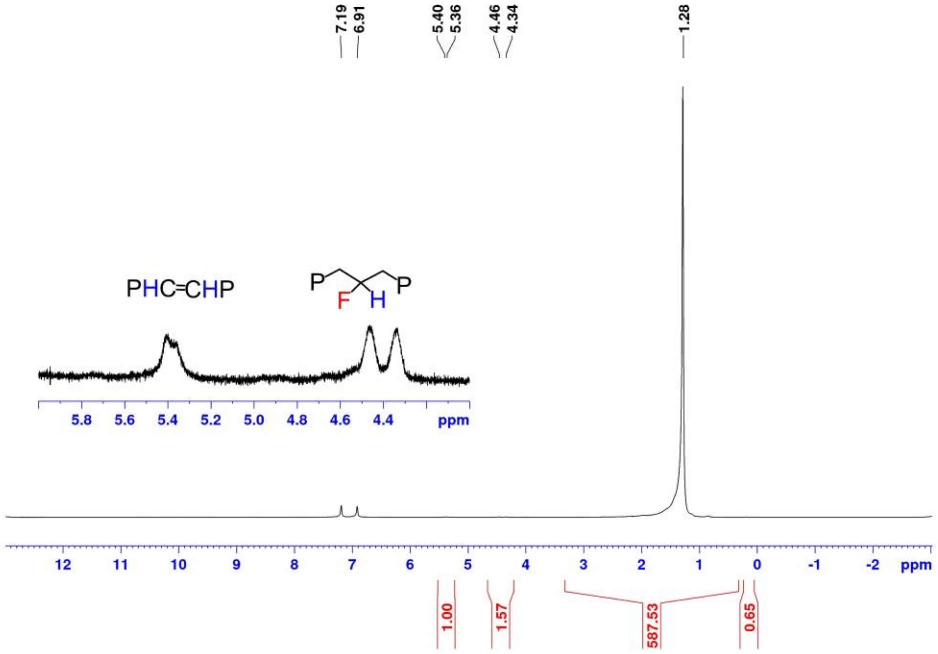

(b) $^{19}F\{^{1}H\}$ (*o*-dichlorobenzene-d_4, 100 ˚C, 470 MHz):

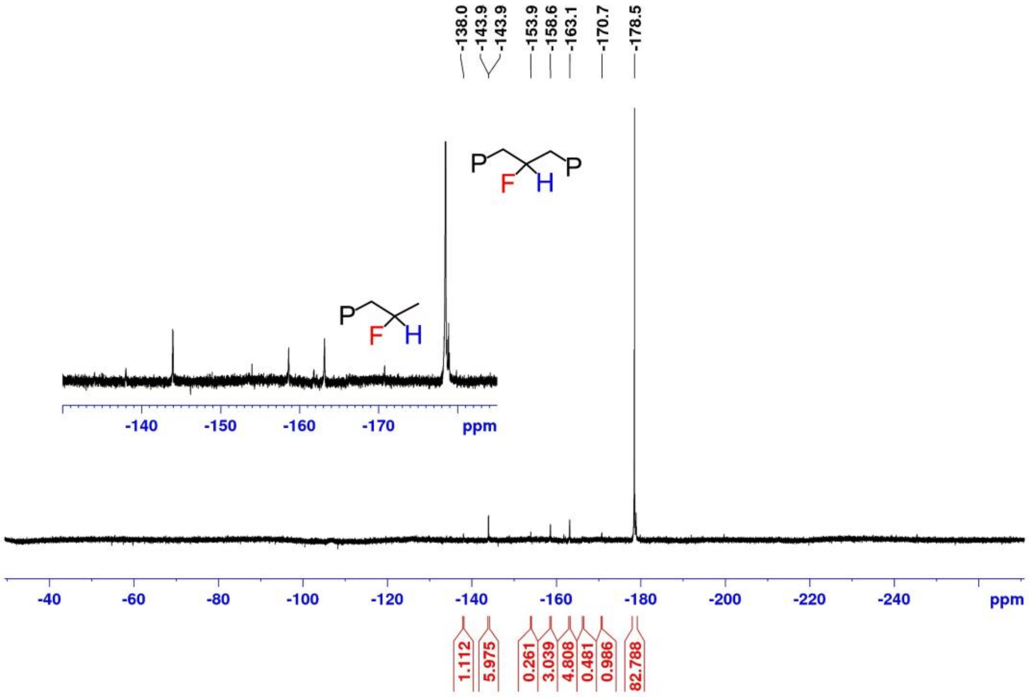

IV. References

1. Liu, Q.; Jordan, R. F., Sterically Controlled Self-Assembly of a Robust Multinuclear Palladium Catalyst for Ethylene Polymerization. *J. Am. Chem. Soc.* **2019**, *141*, 6827–6831.

2. Liu, Q.; Jordan, R. F., Multinuclear Palladium Olefin Polymerization Catalysts Based on Self-Assembled Zinc Phosphonate Cages. *Organometallics*, **2018**, *37*, 4664-4674.

3. Grinshpun, V.; Rudin, A., Measurement of Mark-Houwink constants by size exclusion chromatography with a low angle laser light scattering detector. *Die Makromolekulare Chemie, Rapid Communications* **1985**, *6*, 219-223.

4. Geier, S. J.; Gille, A. L.; Gilbert, T. M.; Stephan, D. W. From Classical Adducts to Frustrated Lewis Pairs: Steric Effects in the Interactions of Pyridines and $B(C_6F_5)_3$. *Inorg. Chem.* **2009**, *48*, 10466-10474

5. Gel’mbol'dt, V. O.; Ennan, A. A. Pentacoordinate fluorosilicate anions. *Russ. Chem. Rev.* **1989**, *58*, 371.

6. Pevec, A.; Demšar, A. The variations in hydrogen bonding in hexafluorosilicate salts of protonated methyl substituted pyridines and tetramethylethylenediamine. *J. Fluor. Chem.* **2008**, *129*, 707.

7. Conley, B. D.; Yearwood, B. C.; Parkin, S.; Atwood, D. A. Ammonium hexafluorosilicate salts. *J Fluor Chem* **2002**, *115*, 155.

8. Christe, K. O.; Wilson, W. W. Reaction of the fluoride anion with acetonitrile. Chloroform and methylene chloride. *J. Fluor. Chem.* **1990**, *47*, 117.

9. Christe, K. O.; Wilson, W. W. Reaction of the fluoride anion with acetonitrile. Chloroform and methylene chloride. *J. Fluor. Chem.* **1990**, *46*, 339.

10. Massey, A. G.; Park, A. J. Perfluorophenyl derivatives of the elements: VII. further studies on tris(pentafluorophenyl)boron. *J. Organomet. Chem.* **1966**, *5*, 218.
